# Evolution of Action Potential Alternans in Rabbit Heart during Acute Regional Ischemia

**DOI:** 10.1155/2015/951704

**Published:** 2015-02-26

**Authors:** Irma Martišienė, Jonas Jurevičius, Rūta Vosyliūtė, Antanas Navalinskas, Rimantas Treinys, Regina Mačianskienė, Rimantas Benetis, Arvydas Matiukas, Arkady M. Pertsov

**Affiliations:** ^1^Laboratory of Membrane Biophysics, Institute of Cardiology, Lithuanian University of Health Sciences, LT-50009 Kaunas, Lithuania; ^2^Department of Pharmacology, SUNY Upstate Medical University, Syracuse, NY 13210-2375, USA

## Abstract

This study investigates the development of the spatiotemporal pattern of action potential alternans during acute regional ischemia. Experiments were carried out in isolated Langendorff-perfused rabbit heart using a combination of optical mapping and microelectrode recordings. The alternans pattern significantly changed over time and had a biphasic character reaching maximum at 6–9 min after occlusion. Phase I (3–11 minutes of ischemia) is characterized by rapid increase in the alternans magnitude and expansion of the alternans territory. Phase I is followed by gradual decline of alternans (Phase II) in both magnitude and territory. During both phases we observed significant beat-to-beat variations of the optical action potential amplitude (OAPA) alternans. Simultaneous microelectrode recordings from subepicardial and subendocardial layers showed that OAPA alternans coincided with intramural 2 : 1 conduction blocks. Our findings are consistent with the modeling studies predicting that during acute regional ischemia alternans can be driven by 2 : 1 conduction blocks in the ischemic region.

## 1. Introduction

Beat-to-beat alternation of the action potential at increased heart rates is extensively used as an empirical midterm and long-term ECG-predictor of ventricular arrhythmias and sudden cardiac death [1–3]. Recently there has been increased interest towards repolarization alternans as a short-term predictor of cardiac arrhythmias and its utilization for antiarrhythmic therapy [4, 5]. Multiple clinical studies have demonstrated upsurge in T-wave alternans and repolarization heterogeneity precede spontaneous initiation of ventricular tachyarrhythmias in humans [6, 7]. In this context, acute regional ischemia, which is known to cause rapid development of conduction abnormalities and arrhythmias, is a useful model for investigating the link between these abnormalities and alternans at short time scales.

Early studies have reported different manifestations of electrical alternans during acute regional ischemia and their connection to dangerous arrhythmias [8, 9]. Yet, technological capabilities at that time were limited to a small number of electrical recordings, which significantly complicated the experimental investigation of this link. The goal of our study is to overcome these limitations by utilizing optical mapping, the technology, which enables detailed spatiotemporal characterization of action potential alternans and which has been proved particularly to be valuable for the analysis of the alternans' mechanisms [10–13].

Here we focus on the inducibility and magnitude of action potential alternans during early stages of acute regional ischemia (<15 minutes), which is characterized by increased probability of spontaneous ventricular tachycardia and fibrillation in various species. The optical mapping is used in combination with microelectrode recordings. The use of microelectrodes enabled the detection of conduction abnormalities occurring deep inside the ventricular wall which are not readily identifiable from the surface recordings and provided new insights with regard to the interpretation of optical recordings from the developing ischemic region and mechanistic links between alternans, conduction blocks, and arrhythmias.

## 2. Materials and Methods

New Zealand white rabbits of either gender (3.4 ± 0.4 kg weight, *n* = 11) were used. All the experimental procedures conformed to the European Community guiding principles and were approved by the State Food and Veterinary Service of the Republic of Lithuania and Ethics Committee of the Lithuanian University of Health Sciences. Animals were sedated by intraperitoneal injection of xylazine (10 mg/kg) including heparin (1000 U/kg) for blood clotting prevention. After 20 minutes animals were additionally anesthetized with intravenous injection of ketamine (10 mg/kg) and then thoracotomy was performed. The hearts were quickly excised, cannulated through the aorta, and attached to a Langendorff perfusion system. To limit the motion, the posterior wall of the heart was pressed against an elastic mesh. The perfusion was carried out under constant pressure (80 mm Hg) with oxygenated (100% O_2_) Tyrode solution (in mmol/L: 135 NaCl, 5.4 KCl, 1.8 CaCl_2_, 0.9 MgCl_2_, 0.33 NaH_2_PO_4_, 10 glucose, 10 HEPES, and pH 7.4 adjusted with NaOH) at 37 ± 0.5°C. To prevent temperature loss, the left ventricular chamber was also perfused with the same Tyrode solution delivered via a tube inserted through a cut in the left atrial appendage.

After 30 minutes of equilibration, the perfusion was switched to a recirculation mode. Blebbistatin (20 *μ*mol/L) with 2,3-butanedione monoxime (BDM, 5 mmol/L) was added to perfusate in order to stop contractions. The hearts were stained with a 10 mL bolus injection of voltage-sensitive dye di-4-ANBDQBS (50 *μ*mol/L) into the perfusate. To increase staining efficiency, we reduced the perfusion rate to 50% of its normal value during dye loading (for 2-3 minutes).

### 2.1. Pacing

The hearts were continuously paced from the endocardial surface of the left ventricular wall via a bipolar silver electrode with 2 ms stimuli at twice the diastolic threshold. The basic pacing cycle length (CL) was set at 300 ms.

### 2.2. Optical Recordings

The optical mapping setup was as described previously [14]. Briefly, fluorescence imaging was carried out using a cooled fast 14-bit EMCCD camera (iXon^EM+^ DU-860, Andor Technology) equipped with 50 mm focal length objective. A continuous-wave, 660 nm, 600 mW diode laser (SDL-660-600T, Shanghai Dream Lasers Technology) with diffuser was used for dye excitation. A 715 nm long-pass filter (NT46-066, Edmund Optics) was placed in front of the camera in order to separate the voltage-dependent fluorescent signal.

**Figure 1:**
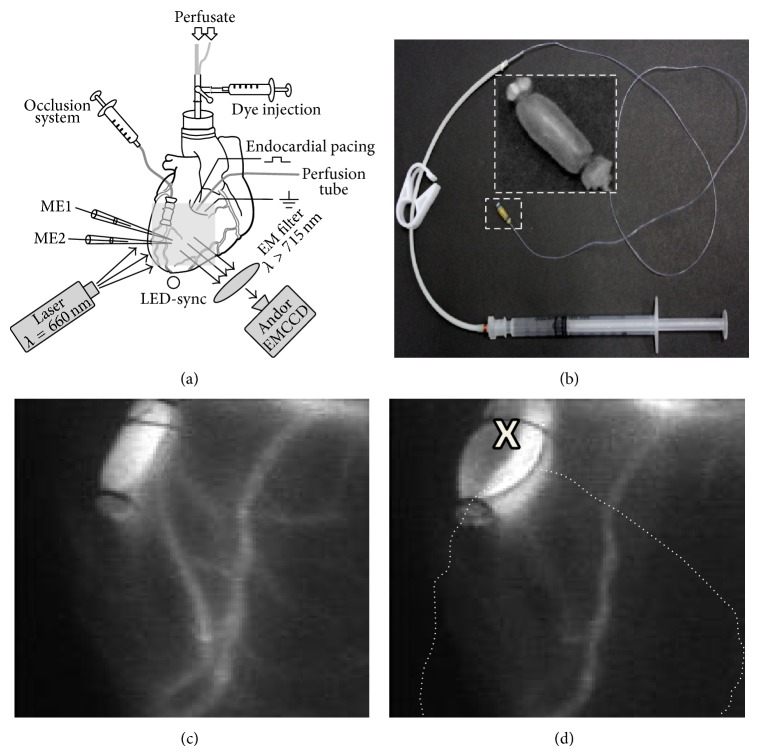
Heart instrumentation. (a) Schematic view of the experimental setup. ME1 and ME2—glass microelectrodes impaled to subendo- and subepicardium, respectively. Grey square on the heart indicates the field of view of the camera. EM filter—emission filter. (b) Custom made balloon occluder made of an inflatable silicon tube (shown at higher magnification in the inset) connected to a syringe. (c) Fluorescent image of the coronary circulation after injection of tetramethylrhodamine-isothiocyanate-Dextran (MW40.000) into the aorta. (d) Image of the same area after occlusion. The “x” indicates the artery occlusion site; the white dotted line shows the boundary of the ischemic region.

We imaged the epicardial surface of the anterior wall of left ventricle. The mapping field was 20 × 20 mm and included ischemic and nonischemic areas (Figure 1(a)). Optical movies were acquired at a frame rate of 500 Hz with a resolution of 128 × 128 pixels using the imaging software (Andor SOLIS x-3467). To mark stimulation time in optical recordings, a small 940 nm LED (light emitting diode) generating 0.5 ms pulses in synchrony with the pacing cycle was placed in the field of view of the camera. The movies were preprocessed using ImageJ 1.45S software. Three-point triangular time and 5 × 5 pyramidal kernels space filters were used; background fluorescence was subtracted from every frame of the recording.

### 2.3. Microelectrode Recordings

We used 2 glass microelectrodes (ME1 and ME2 in Figure 1(a)) filled with 3 mol/L KCl. The depth of impalement was controlled by hydraulic micromanipulators. Action potentials were amplified using a MEZ-7101 amplifier (Nihon Kohden, Japan) and digitized by the 16-channel PowerLab system (ADInstruments) at a frequency of 10 kHz. The data were recorded and analyzed using LabChart8 Pro software.

### 2.4. Data Analysis

The movies of voltage-sensitive fluorescence signals were used to construct isochronal maps, optical action potential duration (OAPD) maps, and alternans maps. The latter included optical action potential amplitude (OAPA), upstroke duration, and OAPD alternans maps. The maps were constructed using custom Scroll 1.16 software developed by Dr. S. Mironov (University of Michigan). The activation time for isochronal maps and OAP duration were evaluated at 50% of depolarization and repolarization levels, respectively. The alternans magnitudes (ΔOAPA, ΔOAPD), were calculated as ΔOAPA = OAPA_n_ − OAPA_n+1_ and ΔOAPD = OAPD_n_ − OAPD_n+1_, where *n* is the beat number. Upstroke duration alternans was calculated in the same manner. ΔOAPA was normalized with respect to OAPA at a given pixel in control.

The alternans area was defined as follows. We counted all pixels that meet the criterion (ΔOAPA/OAPA) ≥ 0.5(ΔOAPA/OAPA)_max⁡_ where (ΔOAPA/OAPA)_max⁡_ is the absolute maximum ΔOAPA/OAPA across the entire mapping area. The resulting number was normalized to the total number of pixels inside the ischemic region. The latter is defined below in Section 2.6 (see Figure 1(d)). The OAPD alternans' area was defined in a similar way.

### 2.5. Statistics

Data are presented as mean ± SEM. Statistical significance between magnitudes of alternans in every ischemia period was evaluated using the Mann-Whitney test; the difference was considered significant when *P* < 0.05. The nonparametric test was chosen since some of our data did not pass the D'Agostino and Pearson omnibus normality test.

### 2.6. Regional Ischemia

To produce acute regional ischemia we occluded the left anterior descending coronary artery (LAD) using a custom made inflatable balloon occluder (Figure 1(b)). Made of an elastic silicon tube, the occluder was gently tied above coronary artery (Figure 1(c)) and inflated via an attached 2 mL syringe. To check the flow before and after occlusion we visualized the coronary vessels by injecting a bolus of fluorescent tetramethylrhodamine-isothiocyanate-Dextran, MW40.000 (7 mg/10 mL, excitation 532 nm, emission 580 nm) into the aorta.

Figure 1(c) shows an image of the major coronary vessels in control conditions (the tracer was injected before LAD occlusion). Figure 1(d) shows the same area after balloon inflation. The tracer does not stain the coronary arteries below the occlusion site (marked “x”) indicating that the flow is fully stopped. To demarcate the ischemic region we used a functional criterion based on action potential shortening, which is very sensitive to ischemia. The dashed line in Figure 1(d) and subsequent figures delineates the area in which after 10 minutes of ischemia the action potential shortened by more than 50% of maximal shortening in the ischemic core.

## 3. Results

### 3.1. Repolarization and Depolarization Alternans

**Figure 2:**
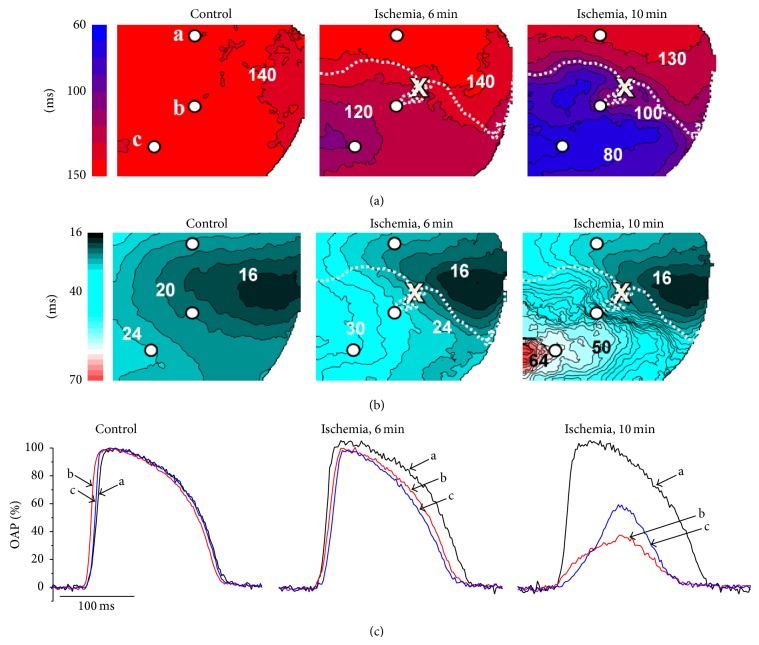
Effect of regional ischemia on optically recorded action potentials in a representative preparation. CL = 300 ms. (a) OAPD maps in control (left), 6 min after occlusion (middle), and 10 min after occlusion (right). Numbers near isolines show APD in ms. Interval between isochrones is 10 ms. The “x” indicates the occlusion site; the white dotted line indicates the boundary of the ischemic zone. (b) Corresponding activation maps. The numbers near isochrones show the activation time in ms. Interval between isochrones is 2 ms. (c) Normalized optical action potentials at 3 different locations indicated as a, b, c (white circles) in (a). In control (left) the action potentials are practically identical. After 6 minutes the action potentials downstream from the occlusion site start shortening (traces b and c), whereas in nonischemic region (trace a) remains unchanged. After 10 minutes of artery occlusion, the action potentials inside the ischemic region show significant deterioration: their amplitude drops and upstroke duration increases which is indicative of developing conduction blocks.

We investigated the evolution of the spatial pattern of the action potential alternans during early stages (<15 minutes) of acute regional ischemia using a combination of optical mapping and intramural microelectrode recordings.  Figure 2 shows OAPD and activation time (isochronal) maps in control (left) and 6 minutes (middle) and 10 minutes after occlusion (right). One can see pronounced shortening of the action potential duration and significant reduction of conduction velocity (“crowding” of isochrones) below the occlusion site (marked “x”) consistent with the advanced regional ischemia. In control experiments without inducing no-flow ischemia, we did not see any changes in action potential duration, propagation velocity, or alternans for at least 30 minutes, which significantly exceeds the duration of our acute ischemia experiments.

**Figure 3:**
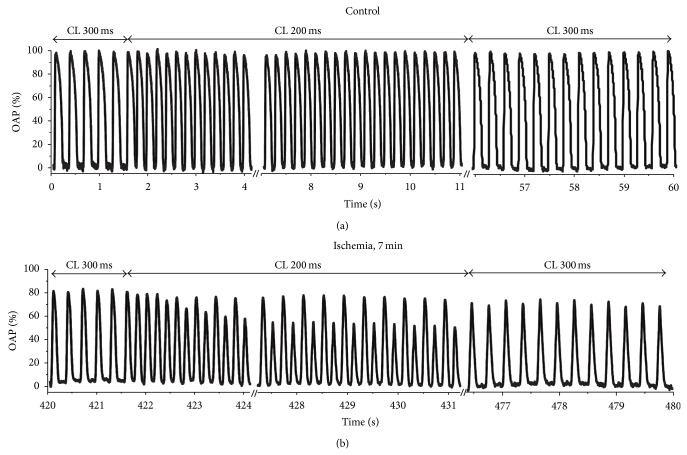
Monitoring the susceptibility to alternans. (a) (Control) OAPs response to abrupt change of CL from 300 ms to 200 ms and subsequent return to 300 ms. Arrows on the top indicate the moments of the CL switch. Small amplitude transient alternans after the switch completely disappears by the 9th second from the beginning of the recording. (b) Recording from the same location at 7 minutes of ischemia. The same pacing protocol initiates significant sustained OAPA alternans at CL = 200 ms.

The susceptibility to alternans during acute regional ischemia and its spatial pattern were determined at 1-minute intervals starting from the onset of coronary occlusion. To achieve this goal, every minute we abruptly reduced the pacing CL from 300 ms to 200 ms and after 10 seconds returned it back to 300 ms.  Figure 3 shows optical action potentials before, during, and after a pacing rate increase in control (panel (a)) and after 7 minutes of ischemia (panel (b)) in a representative preparation. During normal perfusion, we did not observe any manifestations of alternans during the increase in pacing rate (Figure 3(a)). This is consistent with earlier studies in rabbits [11], which reported no alternans at CL > 180 ms during normal perfusion.

The induction of no-flow regional ischemia makes myocardial tissue susceptible to alternans at significantly longer CL than in control. After coronary occlusion, we observed alternans in 8 (out of 11) experiments at CL = 200 ms.  Figure 3(b) shows optical action potentials recorded below the occlusion site during the transition of pacing rate from 300 ms to 200 ms at 7 minutes after the occlusion (the same location as in Figure 3(a)). One can see small beat-to-beat variations in action potential amplitude (APA alternans) at 300 ms which become significantly more pronounced after reducing the pacing interval to 200 ms. The alternans becomes smaller after the CL returns to 300 ms. In 5 (out of 8) experiments we observed ΔOAPA/OAPA = 7.06 ± 0.4% after 6–8 minutes of ischemia at CL = 300 ms.

**Figure 4:**
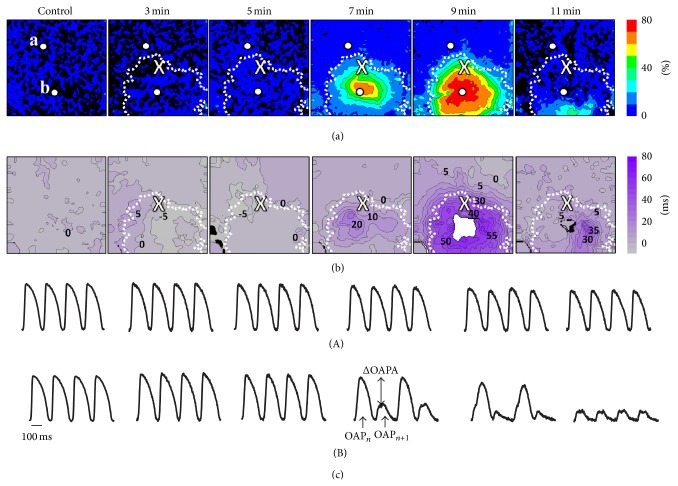
Evolution of OAP alternans pattern during acute regional ischemia. CL = 200 ms. The time after the artery occlusion is shown on top of (a). (a) OAPA alternans maps. Color indicates the normalized value of alternans amplitude (ΔOAPA/OAP_*n*_). Interval between the isolines is 10%. (b) APD alternans maps. The numbers near isochrones show OAPD alternans in ms. The interval between isochrones is 5 ms. White area indicates OAPD alternans >80 ms. (c) Representative OAP recordings from the outside (top row A) and inside (bottom row B) of the ischemic region. The location of the recording sites a and b (white circles) are shown in (a). Other notations are as in Figure 2.

To characterize the spatial and temporal dynamics of the action potential alternans we constructed maps of OAPA and OAPD alternans at different stages of acute regional ischemia.  Figure 4 shows ΔOAPA/OAPA and ΔOAPD maps (Figures 4(a) and 4(b), resp.) in control and at 3, 5, 7, 9, and 11 minutes after occlusion in one of the preparations. The inducibility of the alternans significantly changes over time and has a biphasic character. Phase I was characterized by rapid increase in the magnitude and expansion of the alternans' territory between 3 and 11 minutes of ischemia. Phase I was followed by a gradual decline of alternans' magnitude and territory (Phase II).

The earliest manifestations of alternans are seen in the ΔOAPD maps as early as 3 minutes after occlusion (see Figure 4(b)). This alternans is discordant with relatively small magnitudes. This can be clearly seen from the inspection of the ΔOAPD map in Figure 4(b) which clearly shows both positive and negative values. The maximal values were recorded at the border and outside the developing ischemic region. By minute 7 OAPD alternans becomes concordant and its magnitude significantly increases with the maximum ΔOAPD shifting inside the ischemic region. The increase in magnitude and the spatial expansion of the alternans region persists until minute 9. By then ΔOAPD exceeds 50 ms and the alternans encompasses almost the entire mapping area. As ischemia continues, the susceptibility to alternans starts declining (Phase II): its magnitude becomes smaller and the area it occupies shrinks. In the preparation shown in Figure 4, the effect becomes visible by minute 11. A similar effect was observed in other preparations.

A characteristic feature of electrical alternans during acute regional ischemia is significant changes in OAP amplitude (OAPA alternans). OAPA alternans becomes noticeable after 7 minutes of ischemia. Similar to the OAPD alternans, at 9 minutes the magnitude of OAPA alternans significantly increases and its area expands to occupy the entire ischemic zone. After 11 minutes OAPA alternans amplitude becomes significantly smaller and is noticeable only in a small area near the apex, adjacent to the area of maximal OAPD alternans (compare respective maps in Figures 4(a) and 4(b)). The decline of OAPA alternans correlates with the overall significant reduction of the optical action potential amplitude inside the ischemic region as can be seen from the optical recordings from point b (Figure 4(c), bottom right).

**Figure 5:**
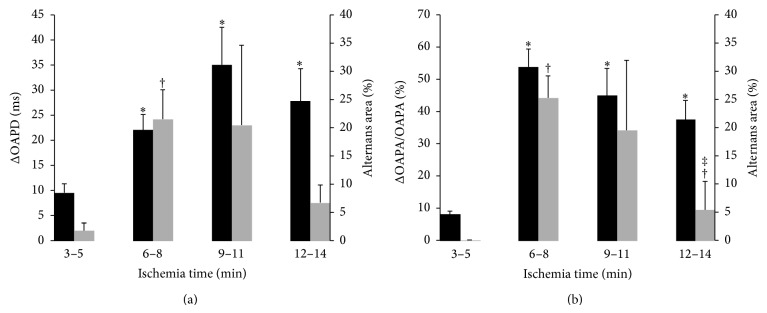
Changes of OAPD (a) and OAPA (b) alternans magnitude and area as a function of time after coronary occlusion. Black and grey columns show the maximum alternans magnitude and area, respectively. ^*^ *P* < 0.05 comparing alternans magnitude to 3–5 min;  ^†^ and ^‡^ *P* < 0.05 comparing alternans area to 3–5 min and to 6–8 min ischemia time, respectively.

The averaged data for all preparations are summarized in Figure 5. The magnitudes of the OAPD alternans (panel (a), black column) become noticeable after 3–5 minutes of ischemia. At this phase, ΔOAPD reaches 10 ms and its area occupies about 2.5% of the ischemic territory (grey column). After 6–8 minutes, the respective number ΔOAPD exceeds 20 ms and the large alternans area occupies more than 20% of the ischemic region. The increase continues until minutes 9–11, after which we see a tendency to decline. The time course of the depolarization or the OAPA alternans (panel (b)) has a similar trend. However, at the earliest stages of ischemia it is less pronounced than OAPD or repolarization alternans. At 3–5 minutes after the ischemia onset, the OAPA alternans is barely detectable occupying less than 1% of the ischemic area.

### 3.2. Depolarization Alternans and Conduction Block

Simultaneous microelectrode recordings obtained at different depths inside the developing ischemic region concurrently with the optical mapping suggest that the depolarization alternans could be caused by intramural conduction blocks. The electrodes were impaled one above the other in the subepicardial (~0.5–1 mm) and subendocardial (~3–5 mm) layers. To enable the registration of the optical and electrical recordings the tips of the electrodes were aligned to have the same projection onto the imaging plane. The impalement site was chosen below the occlusion site inside the ischemic region.

**Figure 6:**
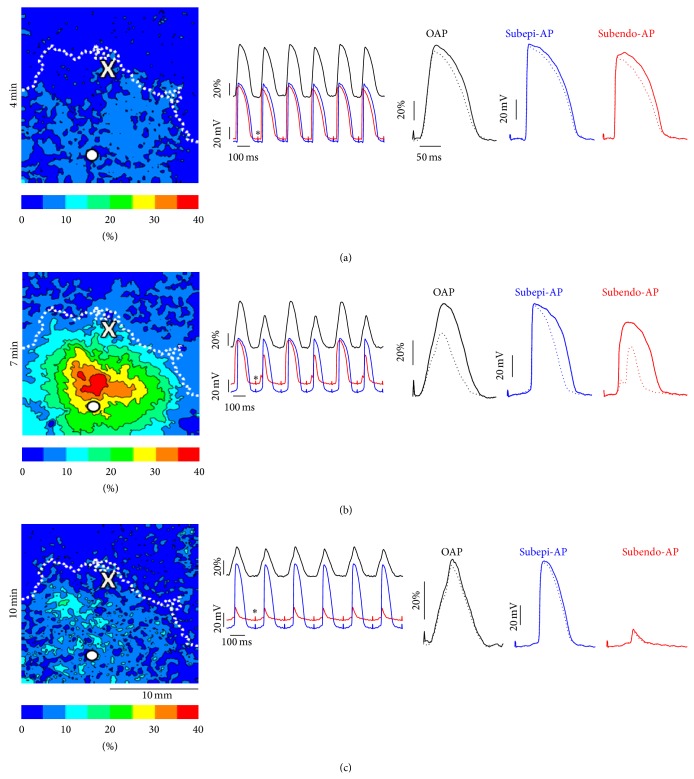
Comparison of simultaneous optical and electrical measurements inside the ischemic region after 4 (a), 7 (b), and 10 minutes (c) of artery occlusion. CL = 200 ms. Left, OAPA alternans maps. Interval between the isolines is 5%. Middle, optical (normalized to control, black trace) and electrical AP's in mV at 0.5 mm (blue trace) and at 3 mm (red trace) recorded simultaneously in the location indicated by the white circle in the OAPA maps. Asterisks show stimulation artifacts. Right, overlapped optical, and electrical AP's of odd (solid trace) and even (round-dotted trace) beats. Other notations are the same as in Figure 2.

Figure 6 compares the results of simultaneous optical and electrical measurements in one of the experiments. The white circle on the OAPA alternans maps (Figure 6, left) indicates the projection of the microelectrode tips onto the epicardial surface. At the fourth minute of ischemia (Figure 6(a)) the optical and microelectrode recordings show similar signals with small (~10%) APA and APD alternans. The situation dramatically changes after seven minutes of ischemia (Figure 6(b)). The optical action potentials acquire a distinct triangular shape with a slow depolarization phase and drastically increased alternans magnitude. The microelectrode recordings now also show very different signals. While the electrode impaled in subepicardium continues to record rather normal propagating action potentials (blue trace), the microelectrode recording from subendocardium provides clear evidence of conduction abnormalities (red trace). The even action potentials have amplitude of less than 40 mV, which cannot represent a propagating response suggesting a 2 : 1 conduction block. After 10 minutes of ischemia the amplitude of the optical signals dropped below 50% of their original value and the OAPA alternans disappeared (Figure 6(c)). At this stage subendocardial microelectrode (red trace) shows full conduction block. Interestingly, the subepicardial electrode continued to display normal propagation without alternans.

**Figure 7:**
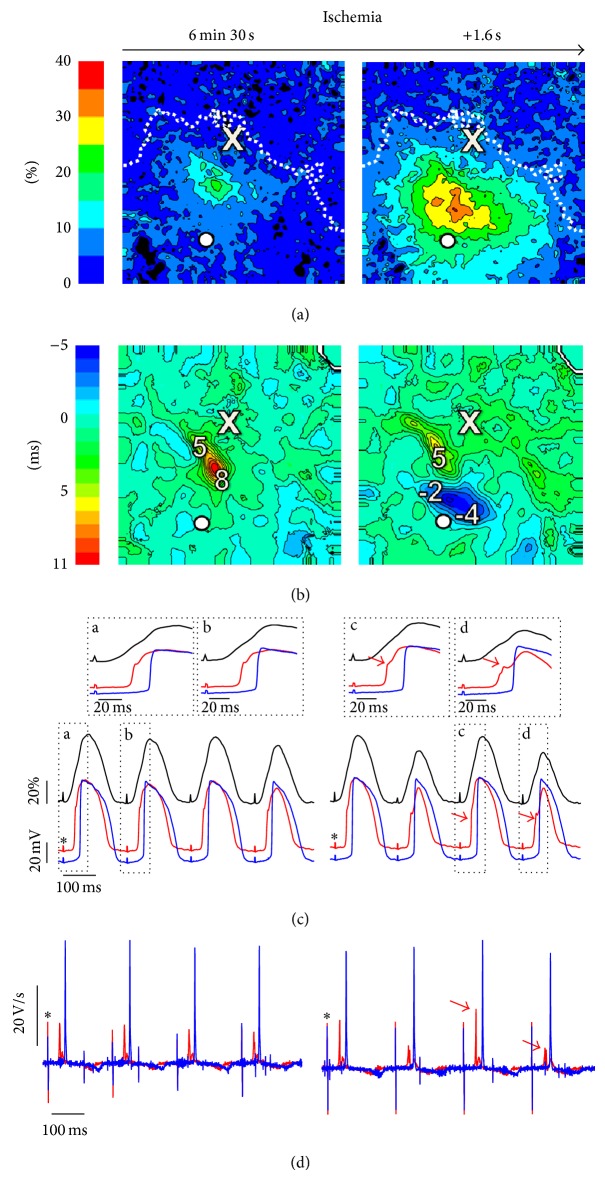
Abrupt onset of OAPA alternans during ischemia. CL = 200 ms. (a) OAPA alternans maps taken at 1.6 seconds interval. (b) Corresponding OAP upstroke duration alternans maps. Interval between the isolines is 1 ms. (c) Superposition of optical (black trace) and microelectrode recordings taken at 0.5 mm (blue trace) and 3 mm (red trace) under the epicardial surface. The recording site is indicated by the white circle in (a). The insets show the upstrokes of optical and electrical action potentials of the odd and even beats at higher time resolution. (d) The first derivative of the transmembrane potential recordings in (c). Red arrows show alternating fragmentation of the depolarization front and alternans of the maximal depolarization rate from the deeper electrode. Asterisks show stimulus artifacts.

The connection between the OAPA alternans and propagation block becomes particularly apparent in the experiments in which the OAPA alternans maps were obtained at increased time resolution.  Figure 7 shows two OAPA alternans maps (panel (a)) separated by 1.6 seconds and obtained after 6 minutes and 30 seconds of ischemia. These recordings indicate that the expansion of the OAPA region occurs abruptly and takes seconds rather than minutes. Notably, this expansion coincides in time with the fragmentation of the depolarization front in the subendocardial microelectrode recording. The fragmentation was most pronounced in the even beats which is characteristic of 2 : 1 conduction block.

To characterize the development of a conduction block in space, we constructed OAP upstroke duration alternans maps (see Figure 7(b)). It is interesting that the upstroke alternans have abruptly become discordant (the second map contains both positive and negative values). The onset of discordant alternans indicates significantly increased dispersion in conduction. Altogether, the abrupt expansion of the OAPA alternans region coupled with reduced depolarization rates, discordant OAP upstroke alternans, and upstroke fragmentation in microelectrode recordings links OAPA alternans to conduction abnormalities. The link between the development of OAPA alternans and conduction abnormalities was corroborated in all four experiments with simultaneous microelectrode and optical recordings.

### 3.3. Depolarization Alternans and Arrhythmia

In 3 out of 11 experiments we observed ventricular fibrillation after coronary occlusion. The fibrillation occurred after 7.83 ± 1.59 minutes of ischemia and correlated with the onset of the depolarization alternans (see Figure 5). In all cases the fibrillation was triggered by reduction of CL from 300 ms to 200 ms and terminated spontaneously within a few minutes after pacing was stopped.

## 4. Discussion

Our study provides the first spatiotemporal characterization of the evolution of electrical alternans during acute regional ischemia and sheds new light on the mechanisms of ischemia-related electrical alternans. We demonstrate that the alternans' pattern significantly changes over time and had a biphasic character reaching its maximum at 6–9 min after occlusion. Starting from the 6th minute of ischemia, repolarization alternans is accompanied with significant depolarization alternans, or OAPA alternans, which correlates with the development of intramural 2 : 1 conduction blocks.

### 4.1. Electrical Alternans Mechanisms during Acute Regional Ischemia

APD alternans is often linked to the steep slope (>1) of the APD-restitution curve defined as the dependence of the APD on the preceding diastolic interval [1, 3, 15, 16]. It has been shown, however, that during acute ischemia the steepness of the APD-restitution curve declines [12], which makes this mechanism less likely to be responsible for the increase in alternans susceptibility that we observed. Moreover, alternans occurred at relatively long pacing intervals (200 ms) where the APD-restitution curve is rather flat [11, 12], which would be even more difficult to explain by the conventional mechanism determined exclusively by the steepness of the APD-restitution curve.

The analysis of mathematical models suggests that alternans during acute regional ischemia may have a different mechanism [17]. Simulations show that the formation of an ischemic core increases the propensity to 2 : 1 conduction blocks at longer pacing intervals. Moreover, when such alternating blocks develop, they drive the discordant APD alternans at the periphery of the 2 : 1 block area. This particular alternans mechanism, called “electrotonic,” is different from other repolarization alternans mechanisms. The area of block serves as a current sink, which drains current shortening the APD in the adjacent normal tissue every other beat.

Our experimental observations are consistent with the model predictions. Between 6 and 11 minutes of ischemia we observed significant reduction in optical action potential amplitude inside the ischemic region suggesting the development of an unexcitable ischemic core inside the ventricular wall (see next section for more details). Microelectrode recordings obtained during this period show significant depolarization and the existence of nonconducting areas. Concurrently we observed the development of significant depolarization or OAPA alternans (see Figure 6). The onset of OAPA alternans is abrupt and simultaneous with the development of the 2 : 1 conduction block as documented by microelectrode recordings (see Figure 7). The development of 2 : 1 block coincides in time with the maximum alternans magnitude which is in agreement with the electrotonic mechanism.

Interestingly, the electrotonic mechanism can explain the experimental observation of discordant alternans of internal Ca transients [18] during early ischemia. Simulations show that as a consequence of electrotonic interaction, the inward Ca current, modulating Ca transients, exhibits discordant alternans at the periphery of the 2 : 1 block area [17]. The model also predicts that at low resting potentials characteristic for the ischemic region the L-type Ca channels can be involved in propagation and affect the development of alternating conduction blocks. Simulations show that slowing the voltage-dependent inactivation of Ca current can result in an earlier onset and larger areas of 2 : 1 conduction block [17]. Altogether this suggests that the “electrotonic” mechanism can play an important role in the alternans phenomena during acute regional ischemia.

### 4.2. OAP Alternans as a Marker of Intramural 2 : 1 Conduction Block

It is well established that an optically recorded action potential represents a weighted sum of the action potentials over a certain interrogation volume [19]. The summation effect is well characterized in multiple theoretical and experimental publications and has been extensively used for the interpretation of optically recorded action potentials in various contexts [20–23]. The main parameter that determines the interrogation volume is the rate of light decay inside the tissue, or so-called attenuation length *δ*. According to the literature, the attenuation length *δ*_*f*_ of the fluorescent light produced by near-infrared voltage-sensitive dye used in this study is >3 mm [24, 25]. It was demonstrated under similar conditions that the layers located 3 mm and less under the surface make substantial contributions to the surface recordings of the optical action potential [24, 25].

**Figure 8:**
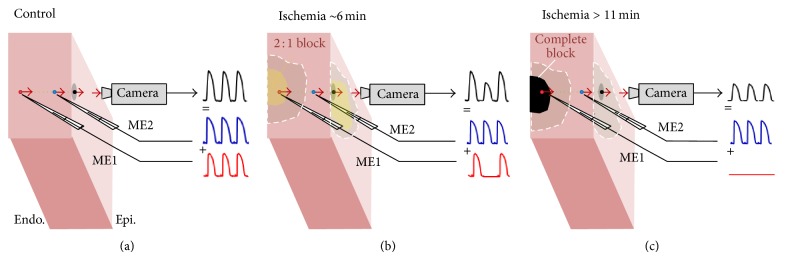
A schematic explaining the evolution of OAPA alternans during acute ischemia and its link with the intramural 2 : 1 conduction block. The traces of the optical and microelectrode recordings (ME1 and ME2) are labeled in black, blue, and red, respectively. The tips of the microelectrodes are located right under the pixel (black point) viewed by the CCD camera. Due to depth averaging effect the optical recording constitutes a weighted sum of signals originating at different depths and represented by ME1 and ME2. (a) Control, all recordings show 1 : 1 conduction and no alternans. (b) After 6 minutes of ischemia an area of 2 : 1 conduction block (shown in yellow) develops inside the ischemic region (shown in grey), while the subepicardial layer preserves normal 1 : 1 conduction. The summation of ME1 and ME2 results in OAPA alternans. (c) As ischemia progresses, the 2 : 1 block evolves into complete conduction block (black region) leading to simultaneous disappearance of the action potential from ME1 and the OAPA alternans.

The summation effect provides a natural explanation of the link between the OAPA alternans and the intramural 2 : 1 conduction block.  Figure 8 illustrates the case when a time-dependent conduction block develops inside the tissue under a normally conducting surface layer. The fact that the tip of the subendocardial microelectrode in our experiments was ~3 mm deep implies that the area of the 2 : 1 conduction block is located within ~3 mm under the surface. Thus, one can safely assume that the area of block is located well inside the interrogation volume. A weighted sum of the signals originating in the epicardial and subendocardial layers (blue and red traces, resp.) would result in nothing else but the OAPA alternans.

A similar analysis can explain the experimentally observed shrinkage of the OAPA alternans' zone with the progression of ischemia.  Figure 8(c) shows an example representing the later stage of ischemia characterized by the presence of a nonconductive domain. In this case, the interrogation volume contains two subvolumes: one with 1 : 1 conduction and a nonconductive one with no changes in the transmembrane potential. The spatial averaging effect in this case would result in low optical signal with no alternans, which is consistent with the experimental observations.

Notably, the link between the OAPA alternans and the 2 : 1 conduction block has been also reported for heart failure models [26, 27]. It is not unlikely that it may have a similar explanation. This may suggest that OAPA alternans is a marker of 2 : 1 conduction block.

### 4.3. Electrical Alternans as a Short-Term Predictor of Arrhythmias during Acute Regional Ischemia

The peak of OAPA and OAPD alternans coincided in time with increased propensity to arrhythmias (see Section 3.3). The arrhythmias were provoked by a mild reduction in CL and in all preparations they occurred after approximately 7 minutes of ischemia. This was not likely to be coincidental given that at this phase of ischemia we also observed the increased incidence of time-dependent conduction blocks, which are necessary for the initiation of reentry.

It is well established in large animal models (pig and dog) that acute regional ischemia can cause spontaneous onset of arrhythmias. The probability of such arrhythmias during acute ischemia increases between 3 and 15 minutes [28]. The highest incidence of ventricular fibrillation is after 5-6 minutes of ischemia. Then it drops down and increases again around 12–30 minutes after coronary occlusion [29]. The earliest arrhythmias are known as type 1a and are attributed to the reentrant mechanism. No such phenomenon, however, was known for smaller animals. Our findings suggest that the rabbit model may not be fundamentally different from the large animal models. The rabbit model of acute regional ischemia may also have a well-defined window with increased propensity to arrhythmia which has yet to be established.

## 5. Conclusions

The inducibility of alternans during acute regional ischemia significantly changes over time and has a biphasic character reaching its maximum between 6 and 11 min of ischemia. During both phases we observed significant optical action potential amplitude (OAPA) alternans. The development of OAPA alternans during acute regional ischemia correlates with the depolarization/conduction alternans. Our findings are consistent with the hypothesis that during acute regional ischemia the alternans can be driven by 2 : 1 conduction block.
